# New insights into the increased risk of migraines from COVID-19 infection and vaccination: a Mendelian randomization study

**DOI:** 10.3389/fneur.2024.1445649

**Published:** 2024-10-25

**Authors:** Jin Yang, Xiaoli Song, Lei Shi, Shuhao Du, Jieying Zhang, Gang Huang, Xuancheng Zhou, Hao Chi, Qian Zhu

**Affiliations:** ^1^First Teaching Hospital of Tianjin University of Traditional Chinese Medicine, Tianjin, China; ^2^National Clinical Research Center for Chinese Medicine Acupuncture and Moxibustion, Tianjin, China; ^3^Clinical Medical College, Southwest Medical University, Luzhou, China

**Keywords:** COVID-19, COVID-19 vaccination, migraine, GWAS, Mendelian randomization study

## Abstract

**Introduction:**

Migraine is a prevalent neurological disorder characterized by recurrent attacks, leading to a substantial global disease burden. Recent observational studies have reported the onset and worsening of migraine following COVID-19 infection and vaccination. However, traditional observational study designs have limitations in controlling for confounding factors, potentially resulting in biased and inconsistent conclusions. To address this, we applied Mendelian randomization (MR) to investigate the causal relationship between COVID-19 infection and vaccination with migraine.

**Methods:**

This study utilized summary-level genome-wide association study (GWAS) data from the GWAS catalog and FinnGen database to evaluate the effects of varying degrees of COVID-19 infection and vaccination on migraine. We employed inverse variance weighted (IVW) fixed-effect and random-effect models as the primary methods for MR analysis, with MR-Egger and other approaches as complementary methods. Sensitivity analyses, including Cochran's Q test, MR-Egger intercept regression, and MR-PRESSO, were conducted to ensure robustness of the results.

**Results:**

Our MR analysis revealed no significant causal association between COVID-19 infection and migraine. However, a significant causal association was found between COVID-19 vaccination and migraine (beta = 0.071, *P* = 0.034). The results were confirmed through a series of sensitivity tests, demonstrating the robustness of the findings.

**Discussion:**

This study provides novel evidence of a significant causal link between COVID-19 vaccination and migraine, while no such association was observed with COVID-19 infection. These findings may have important implications for clinical practice, particularly in planning treatment adjustments and optimizing patient care for individuals with migraines in the context of COVID-19 vaccination.

## 1 Introduction

Migraine is a chronic neurological disorder characterized by recurrent severe unilateral headaches, usually associated with autonomic symptoms, leading to nausea, vomiting, and photophobia. Given its characteristics and associated symptoms, migraine has a substantial economic and social impact, severely affecting patients' quality of life, social activities, and family life (1–3). A 2021 global burden of disease study found that migraine is the third leading cause of age-standardized disability-adjusted life years among all neurological diseases (4). However, the specific triggers and mechanisms of migraine require further research, and preventive treatments can reduce the frequency of migraines and improve quality of life.

COVID-19, also known as severe acute respiratory syndrome coronavirus 2 (SARS-CoV-2), was first identified in December 2019 and declared a global pandemic by the World Health Organization in March 2020 (5). Despite a series of prevention and control measures, the incidence and mortality rates of COVID-19 continue to rise uncontrollably. As of August 2023, the latest statistics from the World Health Organization report that COVID-19 has caused 760 million infections and 6.9 million deaths, posing a significant threat to global health (https://covid19.who). Vaccination is an important measure for preventing and controlling COVID-19, with currently available types including inactivated vaccines, vector vaccines, RNA vaccines, DNA vaccines, live attenuated vaccines, virus-like particle vaccines, and protein subunit vaccines. At the same time, numerous reports of adverse reactions following vaccination have raised concerns about the safety of COVID-19 vaccines (6). Understanding the relationship between varying degrees of COVID-19 infection and vaccination with other diseases helps in formulating disease prevention strategies.

Most observational studies have reported that patients infected with COVID-19 experience headaches, with potential pathophysiological mechanisms including inflammatory processes, direct viral damage, coagulopathy, hypoxemia, and endothelial involvement (7). Innate immune responses associated with viral infections play a significant role, with interferons, interleukins, and tumor necrosis factors, as key elements of innate immunity, proven to exacerbate headaches (8). Additionally, an evaluation using the U.S. Department of Veterans Affairs national healthcare database showed that 1 year after COVID-19 infection, the risk of long-term neurological sequelae increases (9). A multicenter case study from Italy indicates that post-COVID-19 infection headache is a broadly manifested, heterogeneous symptom that may be persistent and severe (10). Meanwhile, a follow-up study from a Spanish headache clinic indicated that the impact of COVID-19 infection and vaccination on migraine worsening is negligible, possibly due to a placebo effect (11). Research results on COVID-19 vaccination and migraine also present some inconsistent views. On one hand, some studies suggest that the risk of migraine increases in patients vaccinated against COVID-19. For example, a six-month follow-up study on COVID-19 vaccination showed that headaches worsened post-vaccination (12). A review and meta-analysis of adverse reactions to COVID-19 vaccination and headache found that the risk of headache doubled within seven days of vaccination, with no differences between vaccine types. This was considered a secondary reaction due to systemic immune response. One-third of the cases had migraine characteristics such as pulsating, phonophobia, and photophobia (13). On the other hand, some studies suggest that there is no significant association between COVID-19 vaccination and migraine, with some even suggesting that vaccination may alleviate migraine symptoms. For example, a clinical case report of eight chronic migraine cases showed significant improvement and relief of symptoms post-vaccination, hypothesizing that the vaccine may improve symptoms by inhibiting pro-inflammatory cytokine synthesis (14). In summary, the impact of COVID-19 infection and vaccination on migraine remains controversial. Moreover, traditional research methods often fail to control for numerous confounding factors that introduce bias into association analyses, necessitating further research on the impact of COVID-19 infection and vaccination on migraine.

MR is an effective tool for identifying causal associations between two disease phenotypes (15–18). Its theoretical foundation is Mendel's laws of inheritance, where genetic variations are distributed uniformly, independently, and randomly during meiosis. MR analysis uses genetic variations as instrumental variables, effectively avoiding the influence of confounding factors and reverse causation on causal estimates. Additionally, the large sample sizes of genome-wide association studies (GWASs) provide substantial statistical power. Previous GWAS studies have demonstrated associations between thousands of genetic variants and various complex diseases, laying the groundwork for the widespread application of MR. Based on the above knowledge, this study will collect summary data from recent large-scale GWAS studies and use two-sample MR analysis to investigate the effects of different levels of COVID-19 infection and vaccination on migraine. Aimed at helping clinicians and headache specialists predict and accordingly adjust treatment strategies to plan treatment adjustments in advance and optimize patient care.

## 2 Materials and methods

### 2.1 Study design

**Figure 1:**
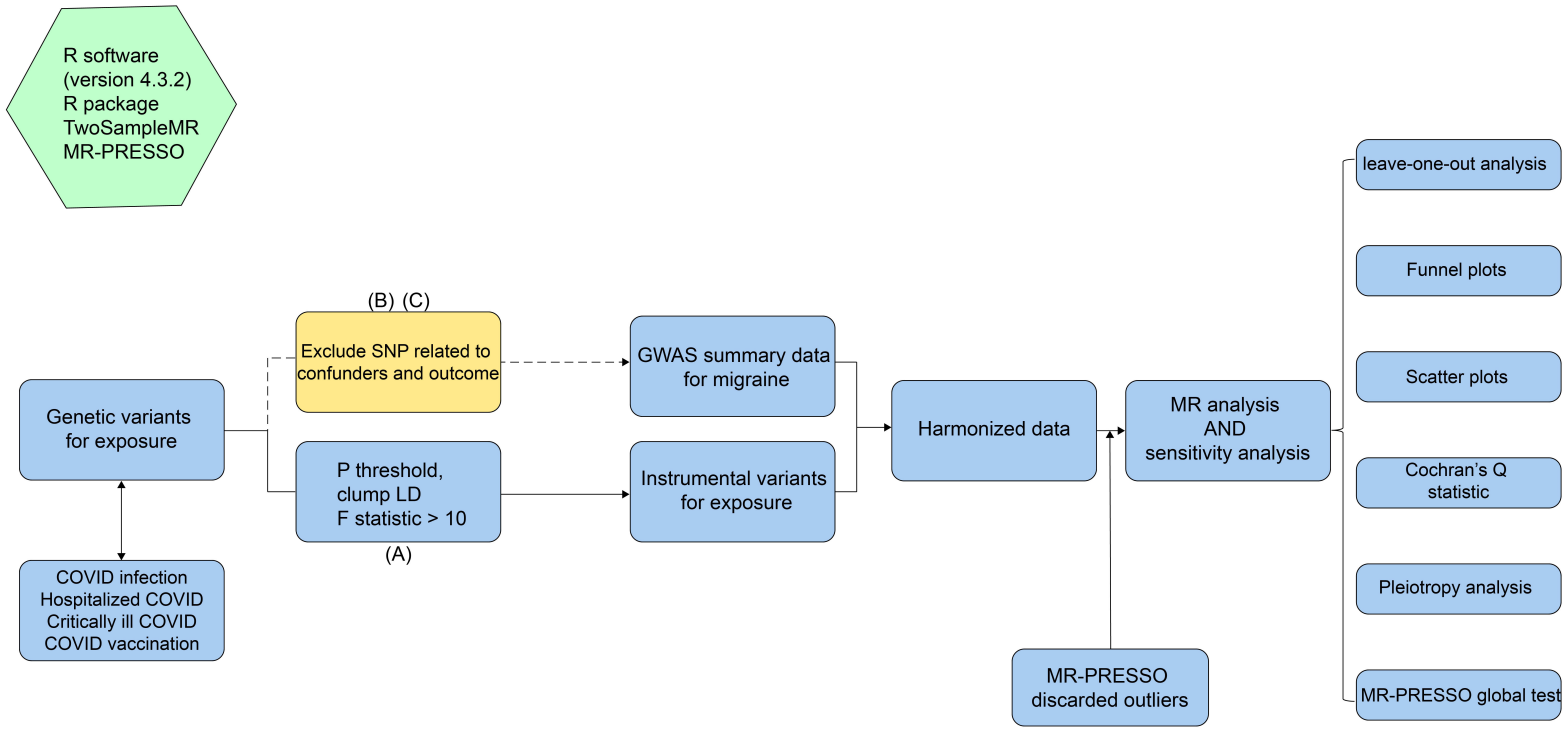
The workflow of this MR study.

The workflow of this MR study is shown in Figure 1. All MR analyses in this study were conducted in strict accordance with the STROBE-MR guidelines (strengthening the reporting of observational studies in epidemiology using Mendelian randomization) (19). The MR analysis was based on summary-level data from large GWAS studies on COVID-19 and migraine, which were publicly available from the GWAS catalog and FinnGen website. We used single nucleotide polymorphisms (SNPs) from genetic variations as instrumental variables (IVs) and applied a two-sample MR approach to investigate the causal effects of varying degrees of COVID-19 infection and vaccination on migraine. Notably, the selection of instrumental variables for MR analysis should adhere to the following three core assumptions: (A) Relevance assumption: IVs are strongly associated with the exposure and can accurately represent the exposure; (B) Independence assumption: IVs are not related to confounders, thus eliminating confounding factors' influence; (C) Exclusion restriction assumption: IVs affect the outcome only through the exposure, with no direct association with the outcome (20).

### 2.2 Data source

Detailed information about data sources is shown in Table 1. Data related to phenotypes of varying degrees of COVID-19 infection were obtained from GWAS studies published in response to the COVID-19 Host Genetics Initiative (21). The phenotypes included general COVID-19 infection with 38,984 cases and 1,644,784 controls; hospitalization due to COVID-19 infection with 8,316 cases and 1,549,095 controls; and confirmed COVID-19 with very severe respiratory infection with 4,792 cases and 1,054,664 controls. Data on COVID-19 vaccination were sourced from the study by Andrea Ganna et al., which included 419,380 samples (22). The inclusion criteria for this study were individuals who had an equal opportunity to receive the first dose of the COVID-19 vaccine by the end of October 2021, excluding those under 30 and over 80 years old, and individuals already diagnosed with COVID-19 before the study. All of the above GWAS studies were conducted on European populations. All data can be publicly accessed in the GWAS catalog (https://www.ebi.ac.uk/gwas/).

**Table 1:** Summary of the genome-wide association studies (GWAS) included in this two-sample MR study.

**Trait**	**Sex**	**Sample size**	**Consortium**	**GWAS ID**	**Population**	**Year**
COVID-19 infection	Males and females	1,683,768	GWAS Catalog	GCST011073	European	2020
Hospitalized COVID-19	Males and females	1,557,411	GWAS Catalog	GCST011083	European	2020
Critically ill COVID-19	Males and females	1,059,456	GWAS Catalog	GCST011078	European	2020
COVID-19 vaccine	Males and females	273,765	GWAS Catalog	GCST90255613	European	2023
Migraine	Males and females	377,277	FinnGen	finngen_R9_MIGRAINE_TRIPTAN	European	2019

The GWAS summary data on migraine were sourced from the FinnGen study, a large-scale genomic study conducted in Finland, covering over 500,000 samples to explore disease mechanisms by associating genetic variations with health data (23). The GWAS ID for migraine is finngen_R9_MIGRAINE_TRIPTAN, including 39,387 cases and 337,890 controls. In the FinnGen database, migraine is defined as having a prescription for a triptan medication and an ICD code for migraine. This data can be publicly accessed from the FinnGen database (https://www.finngen.fi/en/access_results). It is worth noting that all data in this study are publicly available, thus there are no ethical issues involved. Moreover, the COVID-19 related phenotype data and the migraine data come from different GWAS studies, with no known sample overlap.

### 2.3 Selection of instrumental variables

Based on the three core assumptions of MR, we implemented a series of stringent filtering measures on SNPs to obtain high-quality IVs, maximizing statistical power. First, to satisfy the relevance assumption, we used a *p*-value significance threshold of 5E-8 for COVID-19 infection, hospitalized COVID-19, and critically ill COVID-19. Since using 5E-8 as the threshold left too few SNPs for COVID-19 vaccination, we adopted a threshold of 5E-6 to maximize SNP numbers while ensuring statistical power. Additionally, to ensure robust association between SNPs as IVs and COVID-19 related phenotypes, we quantified the strength of each SNP by calculating the F-statistic using the formula F = (β/SE(β))^2^ (24). All SNPs with F-statistics < 10 were excluded from MR analysis to avoid bias from weak instrumental variables. Additionally, we used the online linkage disequilibrium removal function in the TwoSampleMR package with parameters set to R^2^ < 0.001 and kb > 10,000 kb (25). Next, to ensure the independence and exclusivity of SNPs, we used the PheLiGe tool (https://phelige.com) to individually screen the remaining SNPs, removing those associated with outcomes and confounders (*P* < 5E-08, R^2^ > 0.8) to eliminate horizontal pleiotropy bias in MR results (26). Then, we merged the exposure and outcome data, removing palindromic SNPs to ensure that the SNPs in the exposure and outcome data correspond to the same loci. Finally, we used MR-PRESSO to test and remove outlier SNPs to further eliminate horizontal pleiotropy in MR analysis.

### 2.4 Mendelian randomization analysis

In this study, we used five methods to evaluate the effects of various COVID-19 related phenotypes on migraine. IVW fixed-effect and random-effect model results were used as the primary outcome to evaluate the effects of various COVID-19 related phenotypes on migraine (27, 28). Additionally, MR-Egger (29), weighted median (30), weighted mode (31) were used as supplementary references to improve the precision and reliability of the MR analysis. After the analysis, Cochran's Q test was used to check for heterogeneity in the IVW and MR-Egger results (32). If the test result *p* < 0.05 indicated significant heterogeneity, the IVW random-effect model was selected as the final analysis result; otherwise, the IVW fixed-effect model result was used as the final outcome. Notably, regardless of the MR analysis results, the effect size calculations of other methods should be consistent with the IVW method; otherwise, the results are considered not statistically significant (33).

### 2.5 Sensitivity analysis

To further assess the robustness of the analysis results, we conducted systematic sensitivity analyses. First, MR-Egger intercept regression was used to test whether SNPs in the IVs exhibited horizontal pleiotropy and affected the results (34). If the MR-Egger intercept regression analysis showed an intercept value close to zero and *p* > 0.05, it indicated that SNPs in the IVs did not exhibit horizontal pleiotropy. Leave-one-out sensitivity analysis was used to test for heterogeneous SNPs in the IVs by individually removing each SNP and recalculating the overall effect size, comparing changes to determine if MR results were biased by high heterogeneity SNPs (35). Additionally, scatter plots, forest plots, and funnel plots were used to visualize the MR analysis results to check for high-influence SNPs. Meanwhile, to avoid horizontal pleiotropy in the SNPs used as IVs, MR-PRESSO was used to perform a global test for outliers. If the global test result was *P* < 0.05, indicating horizontal pleiotropy, the MR-PRESSO outlier test function was used to identify and remove outlier SNPs (36).

### 2.6 Statistical analysis

The statistical analysis packages used in this study were “TwoSampleMR” and “MR-PRESSO”. All statistical analyses were performed using R version 4.3.2. The significance threshold for *p*-values in MR analysis was set at 0.05, and all statistical tests were two-sided.

## 3 Result

### 3.1 Strength of instrumental variables

In this study, the number of SNPs corresponding to various COVID-19 related phenotypes in MR analysis for migraine is shown in Table 2. Statistical calculations showed that the F-statistics of all IVs were >20. Additionally, there were no known overlaps between the various COVID-19 related phenotype GWAS cohorts and the migraine GWAS cohort, indicating that the selected IVs could effectively avoid potential biases from weak instruments. Meanwhile, MR-PRESSO was used to identify and remove outlier SNPs, followed by a global test estimation. Finally, the MR-PRESSO global test results for IVs corresponding to all COVID-19 related phenotypes were >0.05, indicating that MR-PRESSO did not identify horizontal pleiotropy in the selected IVs for MR analysis (Table 2).

**Table 2:** Mendelian randomization (MR) analysis of COVID-19 related phenotypes (COVID-19 infection, hospitalized COVID-19, critically ill COVID-19 and COVID-19 vaccination) exposure with migraine outcome in European population.

**Exposure**	**Methods**	**No. SNPs**	**Beta**	**SE**	**P (MR analysis)**	**F (min)**	**P(Cochran's Q h eterogeneity test)**	**P (MR-Egger intercept test)**	**P(MR-PRESSO global test)**
COVID infection	IVW-FE	6	0.022	0.050	0	32.494	0.707	0.652	0.747
	IVW-MRE		0.022	0.039	0.564				
	MR-Egger		−0.063	0.182	0.748				
	Weight median		0.039	0.061	0.525				
	Weight model		0.051	0.083	0.568				
Hospitalized COVID	IVW-FE	5	0.019	0.023	0	32.802	0.546	0.212	0.539
	IVW-MRE		0.019	0.020	0.347				
	MR-Egger		−0.335	0.225	0.233				
	Weight median		0.004	0.029	0.890				
	Weight model		0.003	0.034	0.925				
Critically ill COVID	IVW-FE	6	0.004	0.016	0	33.651	0.422	0.155	0.501
	IVW-MRE		0.004	0.016	0.781				
	MR-Egger		−0.099	0.061	0.181				
	Weight median		−0.002	0.020	0.941				
	Weight model		−0.001	0.027	0.983				
COVID vaccination	IVW-FE	46	0.071	0.034	0	21.224	0.505	0.193	0.506
	IVW-MRE		0.071	0.033	0.032				
	MR-Egger		0.179	0.088	0.048				
	Weight median		0.056	0.049	0.247				
	Weight model		0.110	0.119	0.362				

MR, Mendelian randomization; IVW, Inverse variance weighted; SNPs, Single nucleotide polymorphisms; F (min), Minimum F-statistic in each group of SNPs.

### 3.2 Causal effects of various COVID-19 related phenotypes on migraine

**Figure 2:**
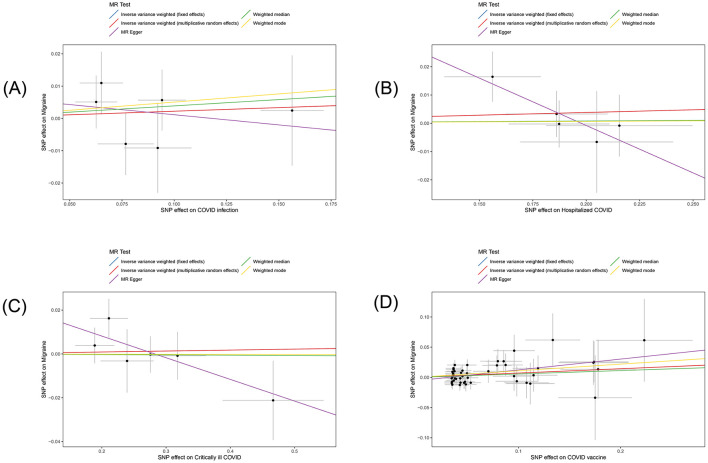
Scatter plot of the estimated effects of COVID-19 related phenotypes (COVID-19 infection, hospitalized COVID-19, critically ill COVID-19 and COVID-19 vaccination) on migraine in the European population through single nucleotide polymorphisms (SNPs). Each black dot represents an individual SNP, plotted with error bars corresponding to each standard error (SE). The slope of each line corresponds to the combined estimates from methods using the weighted fixed effects model of inverse variance (light blue line), weighted random effects model of inverse variance (red line), MR-Egger (purple line), weighted median (green line), and weighted mode (yellow line). **(A)** COVID-19 infection; **(B)** hospitalized COVID-19; **(C)** critically ill COVID-19; **(D)** COVID-19 vaccination.

**Figure 3:**
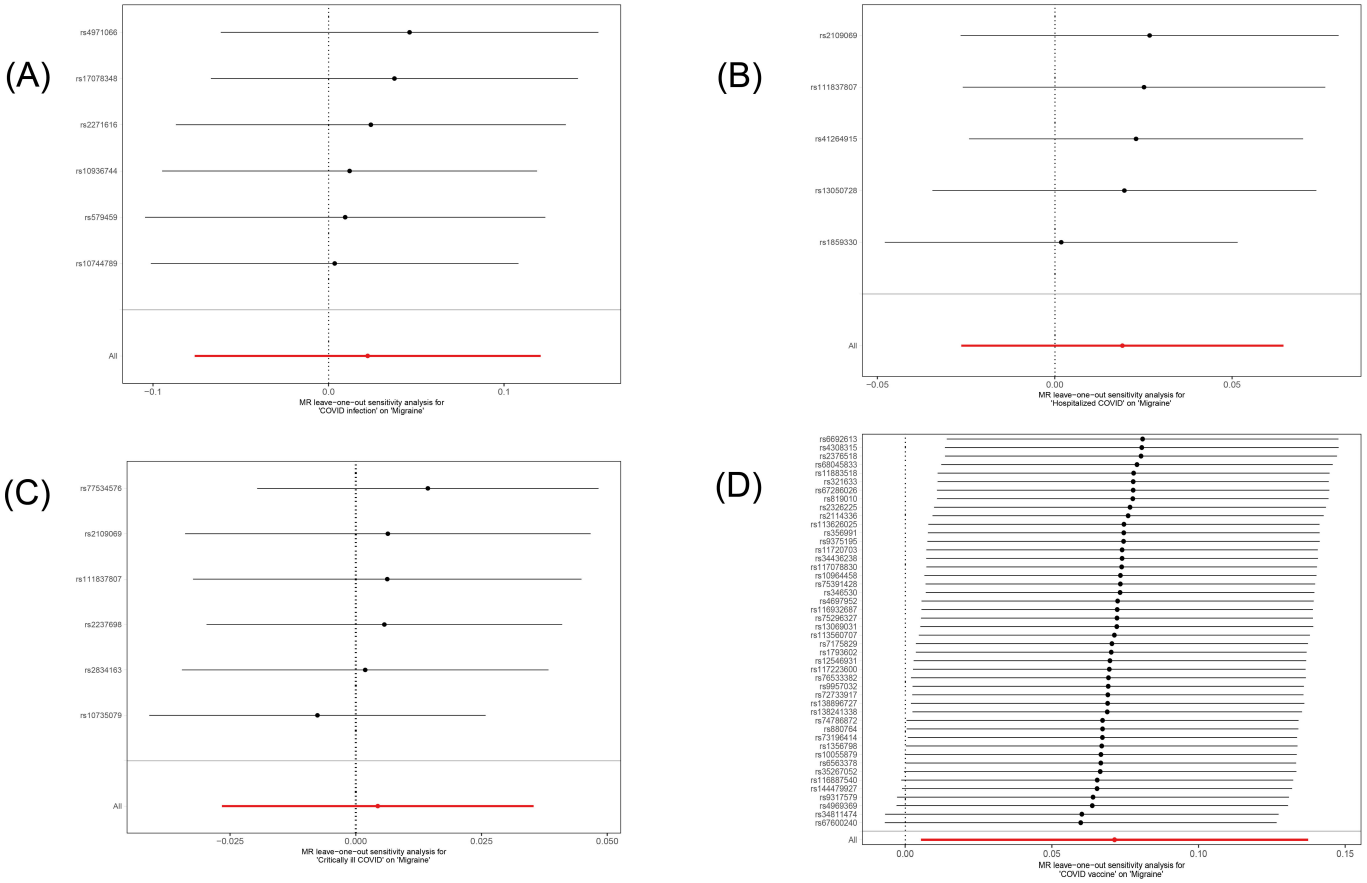
Forest plot of the causal SNP effects of COVID-19 related phenotypes (COVID-19 infection, hospitalized COVID-19, critically ill COVID-19 and COVID-19 vaccination) on migraine in the European population. Error bars represent the 95% confidence intervals (CI). **(A)** COVID-19 infection; **(B)** hospitalized COVID-19; **(C)** critically ill COVID-19; **(D)** COVID-19 vaccination.

In this study, we investigated the effects of COVID-19 infection, hospitalized COVID-19, critically ill COVID-19, and COVID-19 vaccination on migraine. All MR analysis results and sensitivity analysis results are shown in Table 2, with scatter plot visualization of MR results in Figure 2. The Cochran's Q test results for all MR analyses were P>0.05, indicating no heterogeneity, as shown in the funnel plot in Supplementary Figure 1. Therefore, the IVW fixed-effect model was chosen as the primary reference result. Additionally, all MR-Egger intercept regression tests and MR-PRESSO global tests had *P* > 0.05, indicating no horizontal pleiotropy in the MR analysis results, and the results were stable. Finally, through leave-one-out sensitivity analysis, individually excluding SNPs to test their impact on the effect size, no heterogeneous SNPs were identified (Figure 3). Analyzing the MR results, we found that although ordinary COVID-19 infection showed a risk effect in the effect size estimate by the IVW method, *P* > 0.05 did not strongly support this result. Similarly, for the effect estimates of COVID-19 infection leading to hospitalization and critically ill respiratory infection on migraine, all methods had *P* > 0.05, and the effect size estimates from the five methods were inconsistent, providing no sufficient evidence for a significant causal association between these phenotypes and migraine. Notably, when using COVID-19 vaccination as exposure, we found a significant association with migraine (beta = 0.071, *P* = 0.034). Furthermore, all five MR analysis methods showed consistent trends in direction, indicating the robustness of the results.

## 4 Discussion

This study strictly followed the STROBE-MR guidelines to enhance MR analysis, using a two-sample MR approach to investigate the impact of varying degrees of COVID-19 infection and vaccination on migraine. MR analyses were conducted using summary-level GWAS data for COVID-19 infection, hospitalized COVID-19, critically ill COVID-19, and COVID-19 vaccination. All MR analyses adhered to the three core assumptions, and we found that COVID-19 vaccination might increase the risk of migraine (beta = 0.071, *P* = 0.034). Other MR analyses did not yield statistically significant results. In the MR analysis of COVID-19 vaccination on migraine, the estimated effect sizes of the five methods were consistent in direction, with three methods showing statistically significant results. Sensitivity analysis found no heterogeneity or horizontal pleiotropy, proving the robustness and reliability of the MR results.

Our MR study revealed a significant impact of COVID-19 vaccination on migraine. Due to the limited number of previous observational studies on COVID-19 vaccination and migraine, varying quality, small sample sizes, and the difficulty of controlling confounding factors in traditional study designs, results have been inconsistent. An observational study involving 1,819 healthcare workers who received the inactivated virus vaccine found that 30.6% of them experienced headaches after COVID-19 vaccination (37). A case report from Thailand showed that eight patients exhibited characteristic symptoms, predominantly women, with abnormal functional brain imaging without structural changes, as migraine auras after COVID-19 vaccination (38). It was hypothesized that vaccine injection-related pain or vaccine component-related inflammatory reactions might trigger migraine auras in susceptible patients. Although many observational studies have found an association between COVID-19 vaccination and migraine, a few observational studies have different or even opposite conclusions, affecting their persuasive power. For example, a follow-up survey involving 550 participants found no significant difference in headache frequency 1 month before and after infection and vaccination, suggesting that the effects of infection and vaccination on inducing and worsening migraine are negligible (11). Additionally, another case report of eight cases showed that all patients experienced significant improvement and relief of migraine symptoms after vaccination, suggesting that vaccination might alleviate and eliminate headaches by inhibiting the synthesis of pro-inflammatory cytokines (14). However, due to the small sample size and uncontrolled confounding factors, it is likely to produce misleading biases and contradictory conclusions to other observational studies. Traditional observational studies can only identify associations between diseases, not causality, involving a summary of many confounding factors. Therefore, we used a new method, MR, using SNPs as IVs to determine the causal relationship between COVID-19 vaccination and migraine, eliminating the influence of confounding factors to make the results more robust and reliable. Based on this finding, we recommend pre-planning treatment adjustments and optimizing migraine risk management for patients receiving COVID-19 vaccination in clinical practice.

Compared to previous observational studies, our study has the following relative advantages: This is the first time MR analysis has been used to evaluate the impact of varying degrees of COVID-19 infection and vaccination on migraine. Traditional observational study designs have difficulty controlling for numerous confounding factors, which can lead to contradictory results. MR study designs can effectively avoid this issue. Genetic variations are distributed uniformly, independently, and randomly during meiosis, thus effectively avoiding the influence of confounding factors on study results. Additionally, the distribution of genetic variations is determined before the onset of disease, thus effectively avoiding reverse causation (39). The exposure and outcome data analyzed were sourced from two large-scale GWAS studies. The large sample size enhanced statistical power, and the lack of sample overlap between the two studies effectively avoided bias from weak instrumental variables. We conducted a series of stringent sensitivity analyses on the MR analysis results, including MR-Egger intercept regression, leave-one-out sensitivity analysis, Cochran's Q test, and MR-PRESSO global test, to ensure the robustness of the analysis results.

Previous observational studies have shown that COVID-19 infection adversely affects the course of primary headaches, including migraines (40, 41). Results between MR studies and observational studies often show some differences. This may be due to the many unmeasurable confounding factors present in observational studies, such as anxiety, depression, insomnia, unemployment, and lifestyle changes arising from lockdown isolation (42). This could lead to differences between our MR analysis results and previous observational epidemiological studies. Additionally, the reverse placebo effect present in individuals during COVID-19 infection may also be a contributing factor to this observation. The potential cause of migraine due to COVID-19 vaccination may be neuronal damage and immune inflammatory response caused by the vaccination (43). Additionally, individuals may have had an underlying migraine prior to vaccination, which was exacerbated by the vaccination.

Nevertheless, our study still has some limitations. First, our study only established an association between COVID-19 vaccination and migraine, but the underlying mechanisms still need further investigation. Second, the GWAS data used were all based on European populations, limiting the generalizability of the conclusions to other populations. Finally, this MR analysis relied on summary-level GWAS data, thus lacking more detailed clinical information for subgroup analysis.

## 5 Conclusion

In summary, our MR analysis based on European populations found that COVID-19 vaccination alone increases the risk of migraine, and there is no evidence that different levels of COVID-19 infection cause migraine. These findings can help clinicians and headache specialists predict and adjust treatment strategies accordingly, pre-plan treatment adjustments, and optimize patient care. However, our study still has certain limitations regarding the association between different levels of COVID-19 infection and vaccination and migraine. Thus, further rigorous observational studies and comprehensive laboratory research is urgently needed to confirm our conclusions.

## Data Availability

The original contributions presented in the study are included in the article/Supplementary material, further inquiries can be directed to the corresponding author.
