# Supplementary material for: New insights into the increased risk of migraines from COVID-19 infection and vaccination: a Mendelian randomization study
Source: Front Neurol. 2024 Oct 25;15:1445649. doi: 10.3389/fneur.2024.1445649 (PMC11543403; doi:10.3389/fneur.2024.1445649)
Supplement: Supplementary file 1 [file Table_1.DOCX]

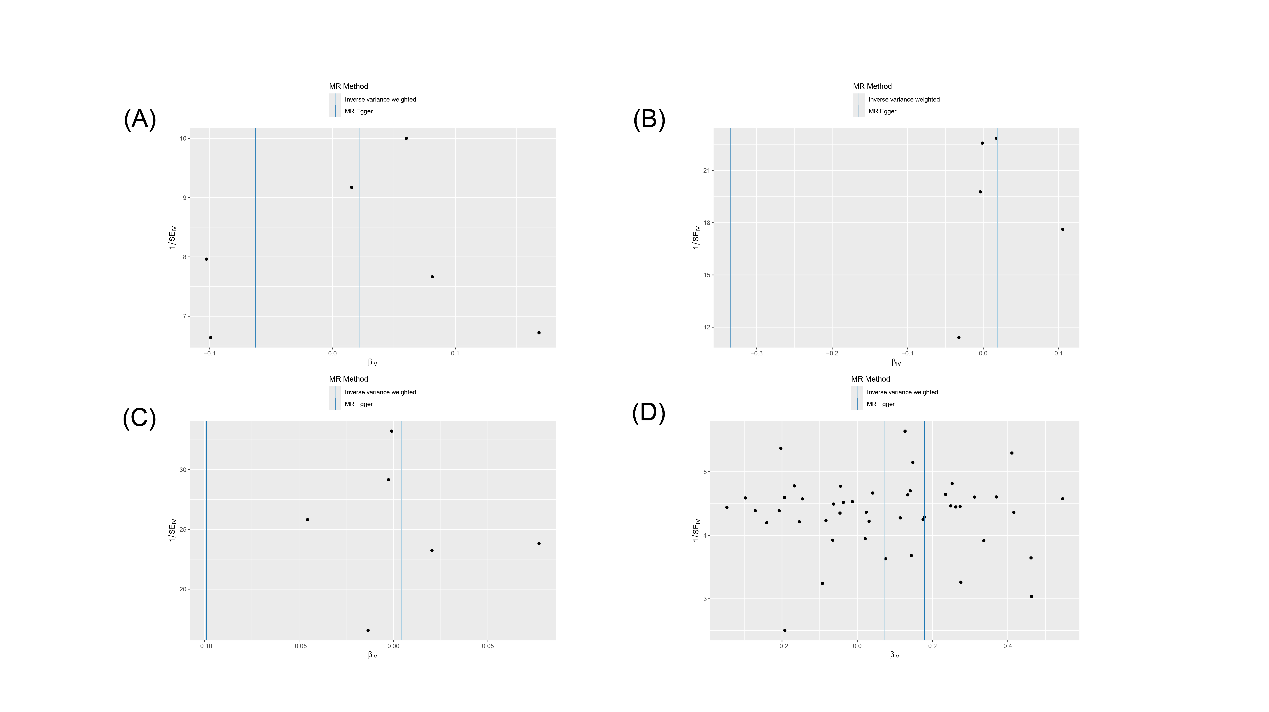


Supplementary Figure 1: Funnel plot showing the relationship between the overall effect estimate of COVID-19 related phenotypes on migraine and the inverse standard error of individual SNP effect estimates. Points represent SNPs, and the two vertical lines indicate the overall effect values calculated by two different methods. The distribution of points reflects the heterogeneity of the analysis. (A) COVID-19 infection; (B) hospitalized COVID-19; (C) critically ill COVID-19; (D) COVID-19 vaccination


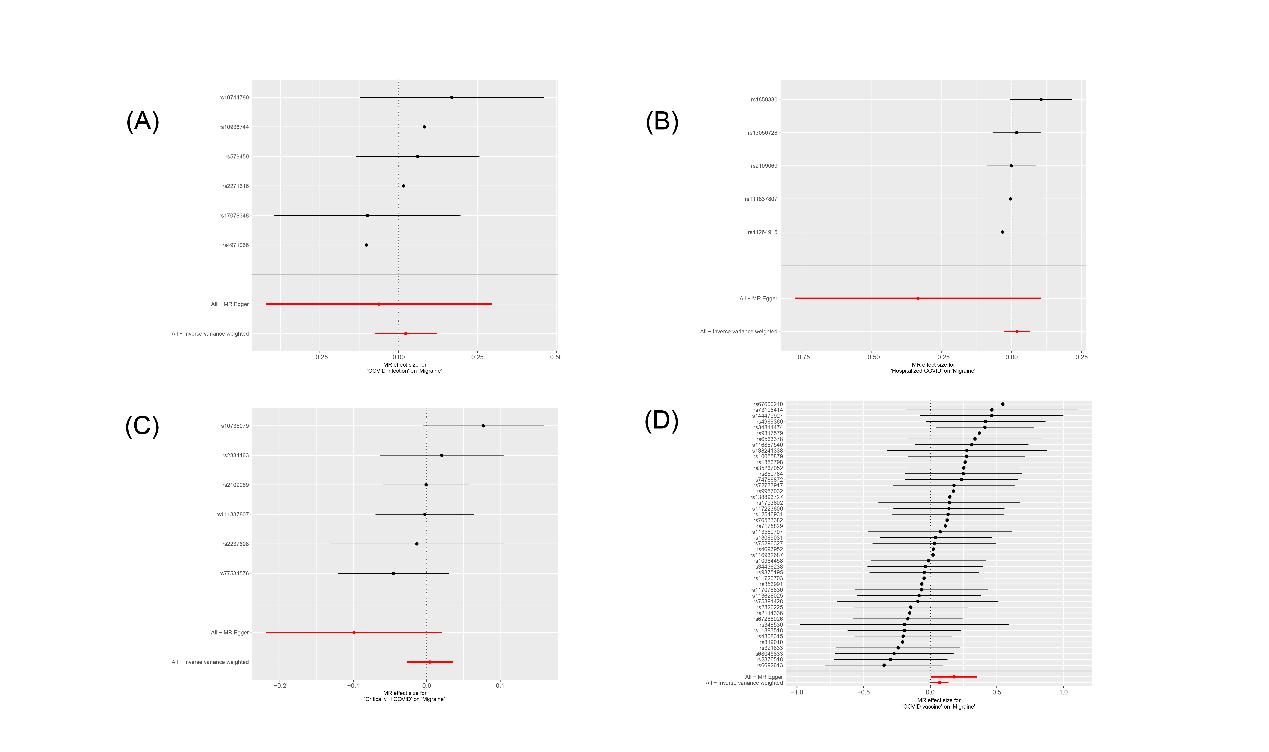


Supplementary Figure 2: Forest plot displaying the effects of individual single nucleotide polymorphism (SNP) associated with COVID-19 related phenotypes and the overall SNP impact on migraine risk. (A) COVID-19 infection; (B) hospitalized COVID-19; (C) critically ill COVID-19; (D) COVID-19 vaccination
